# COVID-19 Disease Course in Former Smokers, Smokers and COPD Patients

**DOI:** 10.3389/fphys.2020.637627

**Published:** 2021-01-27

**Authors:** Ricardo Wesley Alberca, Júlia Cataldo Lima, Emily Araujo de Oliveira, Sarah Cristina Gozzi-Silva, Yasmim Álefe Leuzzi Ramos, Milena Mary de Souza Andrade, Danielle Rosa Beserra, Luana de Mendonça Oliveira, Anna Cláudia Calvielli Castelo Branco, Anna Julia Pietrobon, Nátalli Zanete Pereira, Franciane Mouradian Emidio Teixeira, Iara Grigoletto Fernandes, Alberto José da Silva Duarte, Gil Benard, Maria Notomi Sato

**Affiliations:** ^1^Laboratorio de Dermatologia e Imunodeficiencias (LIM-56), Departamento de Dermatologia, Faculdade de Medicina FMUSP, Universidade de São Paulo, São Paulo, Brazil; ^2^Institute of Biomedical Sciences, University of São Paulo, São Paulo, Brazil

**Keywords:** SARS-CoV-2, COVID-19, infection, smoking, chronic obstructive pulmonary disease

## Abstract

The severe respiratory and systemic disease named coronavirus disease-2019 (COVID-19) is caused by the severe acute respiratory syndrome coronavirus 2 (SARS-CoV-2). Currently, the COVID-19 pandemic presents a huge social and health challenge worldwide. Many different risk factors are associated with disease severity, such as systemic arterial hypertension, diabetes mellitus, obesity, older age, and other co-infections. Other respiratory diseases such as chronic obstructive pulmonary disease (COPD) and smoking are common comorbidities worldwide. Previous investigations have identified among COVID-19 patients smokers and COPD patients, but recent investigations have questioned the higher risk among these populations. Nevertheless, previous reports failed to isolate smokers and COPD patients without other comorbidities. We performed a longitudinal evaluation of the disease course of smokers, former smokers, and COPD patients with COVID-19 without other comorbidities, from hospitalization to hospital discharge. Although no difference between groups was observed during hospital admission, smokers and COPD patients presented an increase in COVID-19-associated inflammatory markers during the disease course in comparison to non-smokers and former smokers. Our results demonstrated that smoking and COPD are risk factors for severe COVID-19 with possible implications for the ongoing pandemic.

## Introduction

Chronic obstructive pulmonary disease (COPD) is a major public health problem, affecting millions worldwide. Common clinical symptoms are dyspnea, cough, and sputum production. The main risk factor for the development of COPD is tobacco smoking, which leads to pulmonary remodeling and inflammation (Marsh et al., 2006). Smoking and COPD are independent risk factors for other diseases such as lung cancer (Biondini et al., 2019) and respiratory infections (Gilca et al., 2011; Aikphaibul et al., 2020; Yoon et al., 2020).

The infection caused by SARS-CoV-2 can lead to the development of a severe pulmonary and systemic disease named COVID-19 (Alberca et al., 2020c). Previous reports have identified that COVID-19 is often more severe in the elderly and individuals with comorbidities such as obesity, hypertension, diabetes Mellitus, other co-infections (Alberca, 2020; Alberca et al., 2020a, d, f; Castelo Branco et al., 2020; Shaw et al., 2020). Those patients generally present an increase in COVID-19-associated inflammatory markers, such as d-dimer, leukocytes count, neutrophil count, neutrophil-to-lymphocyte (NTL) ratio, alanine aminotransferase (ALT), aspartate aminotransferase (AST), and c-reactive protein (CRP) (Wolff et al., 2020). It still not clear the mechanisms that could increase the susceptibility of those groups to a more severe COVID-19, but several mechanisms have been postulated such as dysregulation of the anti-viral immune response (Codo et al., 2020) and increase in the expression of the angiotensin-converting enzyme 2 (ACE2) receptor, SARS-CoV-2 entry receptor (Leung et al., 2020).

Nicotine, a component in tobacco smoke, can suppress antiviral immune responses, via downregulation of Interferon regulatory factor 7 (Han et al., 2019). COPD patients with frequent exacerbations also present a reduction in antiviral immune response, via a reduction in type I and III interferons and interferon-stimulated genes (Singanayagam et al., 2019). In addition, smokers and COPD patients also present an increase in ACE2 receptor expression in the lungs (Leung et al., 2020).

In one of the first reports with COVID-19 patients, 1.1% of the patients had COPD, 12.6% were smokers and 1.9% were former smokers (Guan et al., 2020). COPD and smoking have also been associated with an increased incidence in COVID-19 (Dai et al., 2020; Lian et al., 2020; Zhao et al., 2020) and worst prognoses (Barrasa et al., 2020; Patanavanich and Glantz, 2020).

However, a recent report identified that smoking and pulmonary diseases, such as COPD, were less common in COVID-19 in comparison to influenza patients (Auvinen et al., 2020) and one meta-analyses found no association between COVID-19 prognosis and smoking (Vardavas and Nikitara, 2020).

Nevertheless, to the moment, no study was performed to investigate the difference in the disease course of COVID-19 among smokers, former smokers, and COPD patients without other comorbidities (Dai et al., 2020). Therefore, we aimed to perform an investigation in our cohort to assess if smoking or COPD could influence the COVID-19 disease course.

## Materials and Methods

Patients were recruited at the university hospital (Hospital das Clinicas da Universidade de São Paulo – HCFMUSP), in a special ward for COVID-19 patients. Inclusion criteria were: (1) All patients have SARS-CoV-2 infection verified by nasopharyngeal detection of SARS-CoV-2 by reverse-transcriptase polymerase chain reaction; (2) COPD patients were selected based on previous COPD diagnoses, all patients in our cohort were considered mild to moderate COPD patients; (3) Smoking habits were self-reported by patients on the hospital’s admission. Exclusion criteria were: (1) any other comorbidities other than smoking or COPD; (2) Other co-infection (bacterial or viral). We tracked patients’ clinical laboratory data performed from day 1 at the hospital until SARS-CoV-2 clearance and hospital discharge. This study was approved by the Ethics Committee of HCFMUSP (no. 30800520.7.0000.0068-2020).

In our cohort of 318 patients from the university hospital: Six patients were previously diagnosed with COPD (COPD), seven were active smokers (SMOKERS), and 12 have a previous smoking history (with over 10 years absence) without COPD (EX-SMOKERS), and 10 healthy individuals non-smokers (NC). No patients included in this investigation presented any other comorbidities. Data are shown for the longitudinal graph as median values. Data from the first hospitalization day are shown as median and standard error mean (SEM). Statistical analysis for survival curve was performed using Log-rank test, with Log-rank test for trend and Gehan-Breslow-Wilcoxon test to compare all groups, statistical analysis for other data was performed with Kruskal–Wallis test with Dunn’s multiple comparisons with GraphPad Prism 8 software (GraphPad Software, Inc., San Diego, CA, United States).

## Results and Discussion

The patients did not present any difference in age and hospitalization time was only increased in EX-SMOKERS in comparison to NC (Table 1). On the first hospitalization day, no difference was observed in inflammatory hallmarks of SARS-CoV-2 infection such as lactate dehydrogenase, C-reactive protein, alanine aminotransferase, aspartate aminotransferase, D-dimer, and alkaline phosphatase (Table 1; Alberca et al., 2020b). Previous reports have identified models for predicting the disease outcome based on the first day after hospitalization or based on a risk score (Yildiz et al., 2020). Nevertheless, we hypothesize that longitudinal analysis of clinical data is crucial for the understanding of the immune response to SARS-CoV-2, mainly because it is almost impossible to precisely determine the infection day. Therefore, we performed a daily comparison of clinical data for these patients.

**TABLE 1:** Patients’ characteristics by on the hospitalization day.

	NC (*N* = 10)		EX-SMOKERS (*N* = 12)		SMOKERS (*N* = 7)		COPD (*N* = 6)			
	Mean	SEM	Mean	SEM	Mean	SEM	Mean	SEM	Reference numbers	*p*-value
Age (years)	56.87	3.101	58.43	5.327	60.67	2.909	63.33	3.648	**–**	0.6657
Hospitalization time (days)	18.3*	2.856	37.42*	4.94	32.29	9.411	31.17	5.924	**–**	0.0303
Lactate dehydrogenase (U/L)	**439**	**50.88**	**482.9**	**59.83**	**580.2**	**116.4**	**522.8**	**89.93**	**135–225**	0.5546
C-reactive protein (mg/L)	**184.5**	**43.14**	**143.7**	**45.9**	**191**	**68.06**	**116.7**	**37.54**	**<5.0**	0.7150
Alanine aminotransferase (U/L)	**69.5**	**24.78**	**54.25**	**14**	35.57	8.893	**47.5**	**13.4**	**<41**	0.4593
Aspartate aminotransferase (U/L)	**67.38**	**20.61**	**52.58**	**11.41**	**45.14**	**10.48**	**76.5**	**26.07**	**<37**	0.9193
D-dimer (ng/mL)	**1364**	**307**	**4745**	**2011**	**6042**	**3195**	**3604**	**2286**	**<500**	0.7710
Alkaline phosphatase (U/L)	**221.9**	**47.04**	**185.3**	**51.18**	128.8	30.97	**202.7**	**42.49**	**40–129**	0.3862

*One-way ANOVA with Dunn’s post test. ^∗^ Difference between those groups. References values from Divisão de Laboratório Central to HC/FMUSP. Bold indicates outside reference interval. SEM, Standard Error of Mean; COPD, patients were previously diagnosed with COPD; SMOKERS, active smokers; EX-SMOKERS, patients with a previous smoking history (with over 10 years absence) without COPD; and NC, healthy individuals non-smokers.*

**FIGURE 1:**
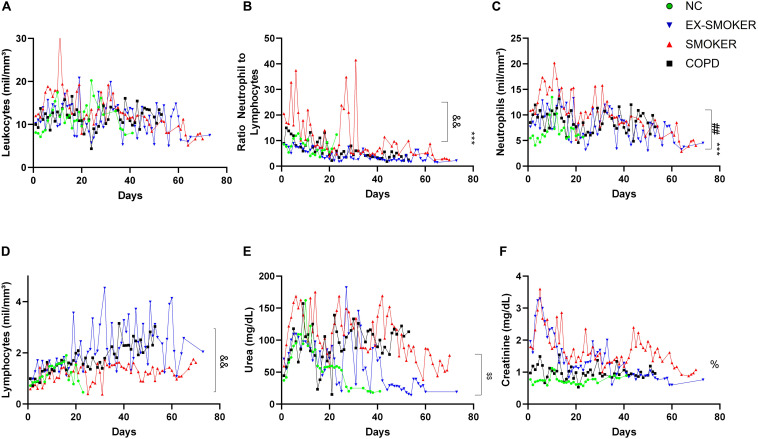
Clinical features of COVID-19 patients. Daily levels of **(A)** Leukocytes, **(B)** Ratio Neutrophil-to-lymphocytes, **(C)** Neutrophils and **(D)** Lymphocytes counts, **(E)** Urea and **(F)** Creatinine levels during the COVID-19 disease course from the first hospitalization day to SARS-CoV-2 clearance and hospital discharge or patients death. NC, No-comorbidities COVID-19 patients; SMOKERS, active smokers by the hospitalization time with COVID-19. EX-SMOKERS, patients that had smoked, but quieted for over 10 years by the hospitalization time with COVID-19; COPD, patients diagnosed with chronic obstructive pulmonary disease prior to COVID-19. *** < 0.001 difference from NC in relation to all other groups. && < 0.01 difference from SMOKER in relation to EX-SMOKERS and COPD. $$ < 0.01 difference from SMOKER and COPD in relation to EX-SMOKERS and NC. ## < 0.01 difference from SMOKER in relation to EX-SMOKERS. % < 0.05 difference among all groups. Kruskal–Wallis test with Dunn’s multiple comparisons. Data collected between 1 March 2020 and 30 August 2020.

During the disease course, no difference in the number of leukocytes was verified among groups (Figure 1A). The NTL ratio was increased in the EX-SMOKERS, SMOKERS, and COPD patients in comparison to NC. It is noteworthy that SMOKERS had a further increase in the NTL in relation to EX-SMOKERS and COPD (Figure 1B). NTL is a widely used maker for COVID-19 prognoses (Alberca et al., 2020b), but the increase in NTL in the SMOKERS groups was due to both an increase in the neutrophil count (Figure 1C) in comparison to EX-SMOKERS and NC and a reduction in the lymphocytes count in relation to EX-SMOKERS and COPD (Figure 1D).

Lymphopenia is a described characteristic in smokers and is linked to worst health prognoses (Biondini et al., 2019). Therefore, smoking may generate immunosuppressive effects, with a reduction in the number of lymphocytes (Düvenci Birben et al., 2016; Tulgar et al., 2016), and consequently a reduction in the anti-SARS-CoV-2 immune response.

In our cohort, COPD and SMOKERS presented increased levels of urea in relation to NC and EX-SMOKERS (Figure 1E). Creatinine levels were different among all groups, indicating that EX-SMOKERS also presented an increase in this inflammatory marker in relation to NC group (Figure 1F). Alterations in the urea and creatinine levels among COPD and Smokers patients could also further indicate an increase in the risk of kidney injury during COVID-19 (Yang et al., 2020).

A report from Wong et al. identified that former smokers present an increased influenza-associated hazard ratio in comparison to non-smokers (Wong et al., 2013). Similarly, in our cohort, the group of EX-SMOKERS presented a difference in the disease course compared to the NC group.

It’s noteworthy that during the investigation two patients from the SMOKERS group passed away due to severe respiratory distress, corroborating with previous reports that identified that smokers with COVID-19 possess a higher odd of developing severe COVID-19. All other patients cleared SARS-CoV-2 infection and were discharged from the hospital. Therefore, in our cohort SMOKERS groups presented a statistically significant difference in the survival curve in comparison to all other groups (*p* = 0.0349).

This phenomenon in smokers and COPD patients could be partially explained by the local inflammatory and oxidative response (Tian et al., 2017) and the up-regulation of ACE2 receptor, SARS-CoV-2 entry receptor, in the lungs (Jacobs et al., 2020). In comparison, allergic asthma downregulates ACE2 receptor expression in the lungs (Castelo Branco et al., 2020), possibly being a protective factor in COVID-19 (Alberca et al., 2020e). This still needs to be further explored since the elderly, an established risk group for severe COVID-19, also presents a downregulation in the ACE2 receptor expression in the lungs (Tavares et al., 2020).

Our report highlights the difference in disease course among smokers, ex-smokers, and no smokers (NC group), indicating that ex-smokers do present a better disease course than smokers. Importantly, we demonstrate that COPD and smoking do influence COVID-19 disease course independently of other comorbidities. Is important to highlight that this investigation possesses a small number of patients and should be further expanded to better understand the influence of these comorbidities on COVID-19. A possible synergic effect of smoking and COPD with other comorbidities in COVID-19 needs to be further explored to aid in the development of specific treatments for these populations.

## Conclusion

In our investigation on the hospitalization day non-smokers, smokers, former smokers, and COPD patients did not present differences in COVID-19 associated inflammatory markers. Nevertheless, a longitudinal investigation demonstrated that smokers and COPD patients, without other comorbidities, present a higher risk for severe COVID-19.

## Data Availability Statement

The original contributions presented in the study are included in the article/supplementary material, further inquiries can be directed to the corresponding author/s.

## Ethics Statement

The studies involving human participants were reviewed and approved by the Ethics Committee of Hospital das Clinicas da Universidade de São Paulo – HCFMUSP (no. 30800520.7.0000.0068-2020). Written informed consent for participation was not required for this study in accordance with the National Legislation and the Institutional Requirements.

## Author Contributions

RA, GB, AD, and MS: conception, analyze the data, and write and review of the manuscript. JL, EO, SG-S, YR, MA, DB, LO, AB, AP, NP, FT, and IF: data collection, analyze the data, and review of the manuscript. All authors contributed to the article and approved the submitted version.

## Conflict of Interest

The authors declare that the research was conducted in the absence of any commercial or financial relationships that could be construed as a potential conflict of interest.

## References

[B1] Aikphaibul P. Theerawit T. Sophonphan J. Wacharachaisurapol N. Jitrungruengnij N. Puthanakit T. (2020). Risk factors of severe hospitalized respiratory syncytial virus infection in tertiary care center in Thailand. *Influenza Other Respi Viruses* 5 64–71. 10.1111/irv.12793 32783380 PMC7767956

[B2] Alberca R. Aoki V. Sato M. (2020a). COVID-19 and HIV: case reports of 2 co-infected patients with different disease courses. *World Acad. Sci. J.* 3:4 10.3892/wasj.2020.75

[B3] Alberca R. W. Andrade M. M. de S. Castelo Branco A. C. C. Pietrobon A. J. Pereira N. Z. (2020b). Frequencies of CD33+ CD11b+ HLA-DR- CD14- CD66b+ and CD33+ CD11b+ HLA-DR- CD14+ CD66b- cells in peripheral blood as severity immune biomarkers in COVID-19. *Front. Med.* 7:580677. 10.3389/FMED.2020.580677 33178720 PMC7592395

[B4] Alberca R. W. Oliveira L. de M. Branco A. C. C. C. Pereira N. Z. Sato M. N. (2020c). Obesity as a risk factor for COVID-19: an overview. *Crit. Rev. Food Sci. Nutr.* 15 1–15. 10.1080/10408398.2020.1775546 32539446

[B5] Alberca R. W. Pereira N. Z. Oliveira L. M. D. S. Gozzi-Silva S. C. Sato M. N. (2020d). Pregnancy, viral infection, and COVID-19. *Front. Immunol.* 11:1672. 10.3389/FIMMU.2020.01672 32733490 PMC7358375

[B6] Alberca R. W. Yendo T. Aoki V. Sato M. N. (2020e). Asthmatic patients and COVID-19: different disease course? *Allergy* 1–2. 10.1111/all.14601 33675252 PMC8250961

[B7] Alberca R. W. Yendo T. M. Leuzzi Ramos Y. Á Fernandes I. G. Oliveira L. M. Teixeira F. M. E. (2020f). Case report: COVID-19 and chagas disease in two coinfected patients. *Am. J. Trop. Med. Hyg.* 103 2353–2356. 33025877 10.4269/ajtmh.20-1185 PMC7695072

[B8] Alberca R. W. (2020). Asthma endotypes and COVID-19. *J. Asthma* 1 1–2. 10.1080/02770903.2020.1825731 32942904

[B9] Auvinen R. Nohynek H. Syrjänen R. Ollgren J. Kerttula T. Mäntylä J. (2020). Comparison of the clinical characteristics and outcomes of hospitalized adult COVID-19 and influenza patients – a prospective observational study. *Infect. Dis. (Auckl)* 10 1–11. 10.1080/23744235.2020.1840623 33170050

[B10] Barrasa H. Rello J. Tejada S. Martín A. Balziskueta G. Vinuesa C. (2020). SARS-CoV-2 in spanish intensive care units: early experience with 15-day survival in Vitoria. *Anaesth Crit. Care Pain. Med.* 39 553–561. 10.1016/j.accpm.2020.04.001 32278670 PMC7144603

[B11] Biondini D. Semenzato U. Bonato M. Tinè M. Bazzan E. Damin M. (2019). Lymphopenia is linked to an increased incidence of cancer in smokers without COPD. *Eur. Respiratory J*. 54:A2586 10.1183/13993003.congress-2019.pa2586

[B12] Castelo Branco A. C. C. Sato M. N. Alberca R. W. (2020). The possible dual role of the ACE2 receptor in asthma and SARS-COV2 infection. *Front. Cell Infect. Microbiol.* 10:550571 10.3389/FCIMB.2020.550571 33072624 PMC7538685

[B13] Codo A. C. Davanzo G. G. Monteiro L. B. Souza G. Muraro S. Carregari V. (2020). Elevated glucose levels favor Sars-Cov-2 infection and monocyte response through a Hif-1α/Glycolysis dependent axis. *SSRN Electron. J.* 10.2139/ssrn.3606770 32864128

[B14] Dai M. Tao L. Chen Z. Tian Z. Guo X. Allen-Gipson D. S. (2020). Influence of cigarettes and alcohol on the severity and death of COVID-19: a multicenter retrospective study in Wuhan. China. *Front. Physiol.* 11:588553. 10.3389/FPHYS.2020.588553 33362576 PMC7756110

[B15] Düvenci Birben Ö Akçay Ş Sezer S. Şirvan Ş Haberal M. (2016). Effect of smoking on peripheral blood lymphocyte subsets of patients with chronic renal failure. *Exp. Clin. Transplant* 14 91–94. 10.6002/ect.tondtdtd2016.P34 27805522

[B16] Gilca R. de Serres G. Boulianne N. Ouhoummane N. Papenburg J. Douville-Fradet M. (2011). Risk factors for hospitalization and severe outcomes of 2009 pandemic H1N1 influenza in Quebec, Canada. *Influenza Other Respi Viruses* 5 247–255. 10.1111/j.1750-2659.2011.00204.x 21651735 PMC4634547

[B17] Guan W. Ni Z. Hu Y. Liang W. Ou C. He J. (2020). Clinical characteristics of coronavirus disease 2019 in China. *N. Engl. J. Med.* 382 1708–1720. 10.1056/NEJMoa2002032 32109013 PMC7092819

[B18] Han H. Huang W. Du W. Shen Q. Yang Z. Li M. D. (2019). Involvement of interferon regulatory factor 7 in nicotine’s suppression of antiviral immune responses. *J. Neuroimmune Pharmacol.* 14 551–564. 10.1007/s11481-019-09845-2 31154625 PMC12360304

[B19] Jacobs M. van Eeckhoutte H. P. Wijnant S. R. A. Janssens W. Brusselle G. G. Joos G. F. (2020). Increased expression of ACE2, the SARS-CoV-2 entry receptor, in alveolar and bronchial epithelium of smokers and COPD subjects. *Eur. Respir. J.* 56:2002378. 10.1183/13993003.02378-2020 32675207 PMC7366177

[B20] Leung J. M. Yang C. X. Tam A. Shaipanich T. Hackett T. L. Singhera G. K. (2020). ACE-2 expression in the small airway epithelia of smokers and COPD patients: implications for COVID-19. *Eur. Respir. J.* 55:2000688. 10.1183/13993003.00688-2020 32269089 PMC7144263

[B21] Lian J. Jin X. Hao S. Jia H. Cai H. Zhang X. (2020). Epidemiological, clinical, and virological characteristics of 465 hospitalized cases of coronavirus disease 2019 (COVID-19) from Zhejiang province in China. *Influenza Other Respi Viruses* 14 564–574. 10.1111/irv.12758 32397011 PMC7273099

[B22] Marsh S. Aldington S. Shirtchiffe P. Weatherall M. Beasley R. (2006). Smoking and COPD: what really are the risks? *Eur. Respir. J.* 28 883–884. 10.1183/09031936.06.00074806 17012635

[B23] Patanavanich R. Glantz S. A. (2020). Smoking is associated with COVID-19 progression: a meta-analysis. *Nicotine Tob Res.* 22 1653–1656. 10.1093/ntr/ntaa082 32399563 PMC7239135

[B24] Shaw P. D. Rao A. P. Malik P. (2020). Cardiac complications of COVID-19: the concealed realities. *Infect. Dis. (Auckl)* 9 1–2. 10.1080/23744235.2020.1841908 33164604

[B25] Singanayagam A. Loo S. L. Calderazzo M. Finney L. J. Torralbo M. B. T. Bakhsoliani E. (2019). Antiviral immunity is impaired in COPD patients with frequent exacerbations. *Am. J. Physiol. - Lung Cell Mol. Physiol.* 317 L893–L903. 10.1152/ajplung.00253.2019 31513433 PMC6962603

[B26] Tavares C. de Am, Avelino-Silva T. J. Benard G. Cardozo F. A. M. Fernandes J. R. (2020). Ace2 expression and risk factors for covid-19 severity in patients with advanced age. *Arq. Bras. Cardiol.* 115 701–707. 10.36660/abc.20200487 33111872 PMC8386971

[B27] Tian Z. Zhang H. DIxon J. Traphagen N. Wyatt T. A. Kharbanda K. (2017). Cigarette smoke impairs A 2A adenosine receptor mediated wound repair through up-regulation of Duox-1 expression. *Sci. Rep.* 7:44405. 10.1038/srep44405 28337995 PMC5364501

[B28] Tulgar Y. K. Cakar S. Tulgar S. Dalkilic O. Cakiroglu B. Uyanik B. S. (2016). The effect of smoking on neutrophil/lymphocyte and platelet/lymphocyte ratio and platelet indices: a retrospective study. *Eur. Rev. Med. Pharmacol. Sci.* 20 3112–3118. 27460742

[B29] Vardavas C. I. Nikitara K. (2020). COVID-19 and smoking: a systematic review of the evidence. *Tob Induc. Dis.* 18:20. 10.18332/tid/119324 32206052 PMC7083240

[B30] Wolff D. Nee S. Hickey N. S. Marschollek M. (2020). Risk factors for Covid-19 severity and fatality: a structured literature review. *Infection* 28 1–14. 10.1007/s15010-020-01509-1 32860214 PMC7453858

[B31] Wong C. M. Yang L. Chan K. P. Chan W. M. Song L. Lai H. K. (2013). Cigarette smoking as a risk factor for influenza-associated mortality: evidence from an elderly cohort. *Influenza Other Respi Viruses* 7 531–539. 10.1111/j.1750-2659.2012.00411.x 22813463 PMC5855151

[B32] Yang X. Jin Y. Li R. Zhang Z. Sun R. Chen D. (2020). Prevalence and impact of acute renal impairment on COVID-19: a systematic review and meta-analysis. *Crit. Care* 24:356. 10.1186/s13054-020-03065-4 32552872 PMC7300374

[B33] Yildiz H. Yombi J. C. Castanares-Zapatero D. (2020). Validation of a risk score to predict patients at risk of critical illness with COVID-19. *Infect. Dis. (Auckl)* 1 1–3. 10.1080/23744235.2020.1823469 33000990

[B34] Yoon J. G. Noh J. Y. Choi W. S. Lee J. Lee J. S. Wie S. H. (2020). A comparison of epidemiology and clinical outcomes between influenza a H1N1pdm09 and H3N2 based on multicenter surveillance from 2014 to 2018 in South Korea. *Influenza Other Respi Viruses* 15 99–109. 10.1111/irv.12795 PMC7767957 32844596

[B35] Zhao Q. Meng M. Kumar R. Wu Y. Huang J. Lian N. (2020). The impact of COPD and smoking history on the severity of Covid-19: a systemic review and meta-analysis. *J. Med. Virol.* 92 1915–1921. 10.1002/jmv.25889 32293753 PMC7262275

